# Single molecule force spectroscopy for evaluating inhibitors of SARS-CoV-2 variants of concern

**DOI:** 10.1007/s00249-026-01818-7

**Published:** 2026-01-19

**Authors:** Rong Zhu, Yoo Jin Oh, Peter Hinterdorfer

**Affiliations:** Department of Experimental Applied Biophysics, Institute of Biophysics, Johannes Kepler University Linz, Gruberstrasse 40, Linz, A4020 Austria

**Keywords:** Atomic force microscopy, Single molecule force spectroscopy, Spike protein, ACE2 receptor, Lectins, Inhibition

## Abstract

The binding between the SARS-CoV-2 Spike protein and its cellular receptor ACE2 is essential for viral entry and infection and, therefore, represents a critical target for antiviral intervention. Single-molecule force spectroscopy (SMFS), conducted using atomic force microscopy (AFM), allows direct investigation of Spike–ACE2 interactions at the single-molecule level, providing insights into binding mechanisms not easily captured by conventional methods. This review discusses recent applications of SMFS to assess molecular inhibitors of Spike–ACE2 interactions, focusing specifically on soluble ACE2 and glycan-binding lectins (Clec4g, hCLEC4G, and engineered H84T-Banlec). These inhibitors demonstrated potent, concentration-dependent activity against multiple SARS-CoV-2 variants, including Delta and Omicron, achieving IC₅₀ values in the low nanomolar range (soluble ACE2: 0.25–0.38 nM; Clec4g/hCLEC4G: 36–58 nM; H84T-Banlec: 2.6–5.2 nM). Notably, single-molecule IC₅₀ measurements closely correlated with EC₅₀ values from cell-based assays, validating the physiological relevance and predictive capability of the SMFS method. Collectively, these findings underscore the utility of AFM-SMFS as a powerful approach for inhibitor evaluation and antiviral discovery, particularly for strategies targeting conserved structural features of the Spike protein to achieve broad-spectrum efficacy against current and emerging viral variants.

**Supplementary Information:**

The online version contains supplementary material available at https://doi.org/10.1007/s00249-026-01818-7.

## Introduction

The initial step in SARS-CoV-2 infection is mediated by high-affinity binding between the viral Spike (S) glycoprotein and the host cell receptor angiotensin-converting enzyme 2 (ACE2), a process that dictates viral entry, tissue tropism, and pathogenicity (Hoffmann et al. 2020; Lan et al. 2020). Accumulating evidence indicates that mutations in the Spike protein—particularly those found in variants of concern (VOCs) such as Alpha, Delta, and Omicron—significantly alter the thermodynamic and mechanical landscape of this interaction, leading to enhanced transmissibility and immune evasion (Liu et al. 2022; Harvey et al. 2021; Zhu et al. 2022).

Given the centrality of the Spike–ACE2 interface in viral entry, it has become a primary target for therapeutic intervention. A range of candidate inhibitors has been explored, including recombinant soluble ACE2 (sACE2) (Monteil et al. 2020), neutralizing antibodies, and glycan-binding lectins that recognize conserved glycosylation motifs on the Spike protein (Monteil et al. 2020; Hoffmann et al. 2021; Barnes et al. 2020; Swanson et al. 2015; Petitjean et al. 2022; Chan et al. 2022). Conventional methods to evaluate such inhibitors—including surface plasmon resonance (SPR), enzyme-linked immunosorbent assay (ELISA), and pseudovirus entry assays—have provided valuable insights into binding affinities and neutralization profiles. However, these ensemble-average methods are inherently limited in their ability to capture molecular heterogeneity, transient intermediate states, and the mechanical environment experienced during receptor engagement (Nie et al. 2020).

Single-molecule force spectroscopy (SMFS) provides a complementary approach that allows direct quantification of unbinding forces and interaction kinetics between individual Spike proteins and ACE2 receptors under near-physiological conditions (Zhu et al. 2022; Yang et al. 2020). In a typical SMFS setup, Spike ectodomains are immobilized onto AFM cantilever tips and brought into contact with ACE2-expressing cells, such as Vero E6. Subsequent retraction of the cantilever results in single bond breakages and enables detection of specific unbinding forces with a few piconewton sensitivity. A key strength of SMFS lies in the precise experimental control it offers. Parameters including pulling velocity, contact time, and inhibitor concentration can be systematically varied. This level of control enables real-time assessment of how inhibitory molecules modulate the strength and frequency of Spike–ACE2 interactions at the single-molecule level. The ability to finely tune these conditions provides a unique window into inhibitory mechanisms that would remain obscured in conventional bulk-phase assays.

AFM-based SMFS complements traditional bulk assays and holds a distinct position among single-molecule methods. Optical tweezers provide adjustable, high-precision forces (Bustamante et al. 2021), magnetic tweezers deliver stable force-clamp measurements over extended time periods (Bauer et al. 2024), and single-molecule FRET reveals conformational dynamics, although it requires fluorescent labeling (Lu et al. 2020). However, these single-molecule techniques are generally limited to purified or reconstituted systems. In contrast, surface-based bulk techniques such as SPR and bio-layer interferometry (BLI) (Yang et al. 2020) yield ensemble kinetic and affinity data but cannot directly resolve single-bond or cell-surface interactions. Taken together, SMFS bridges these methodological gaps by enabling label-free measurement of Spike-ACE2 binding directly on living cell membranes under conditions close to the physiological state, although its implementation requires dedicated chemical functionalization.

In this review, we summarize recent advances in the application of AFM-based SMFS to investigate how molecular inhibitors interfere with the SARS-CoV-2 Spike–receptor interaction at the biophysical level. While AFM-SMFS offers unprecedented resolution at the single-molecule scale, it also has inherent limitations such as the need for specialized instrumentation and lower throughput compared with bulk-phase assays. Nevertheless, when combined with cell-based or ensemble approaches, SMFS provides complementary insights into viral inhibition mechanisms. Particular emphasis is placed on the unique ability of force-resolved, single-molecule analyses to dissect inhibitory mechanisms with a resolution that is often inaccessible to conventional ensemble techniques, thereby offering deeper mechanistic insights into antiviral strategies targeting the virus–host attachment process.

## Binding of Spike to ACE2 expressed in Vero E6 cells

**Fig. 1:**
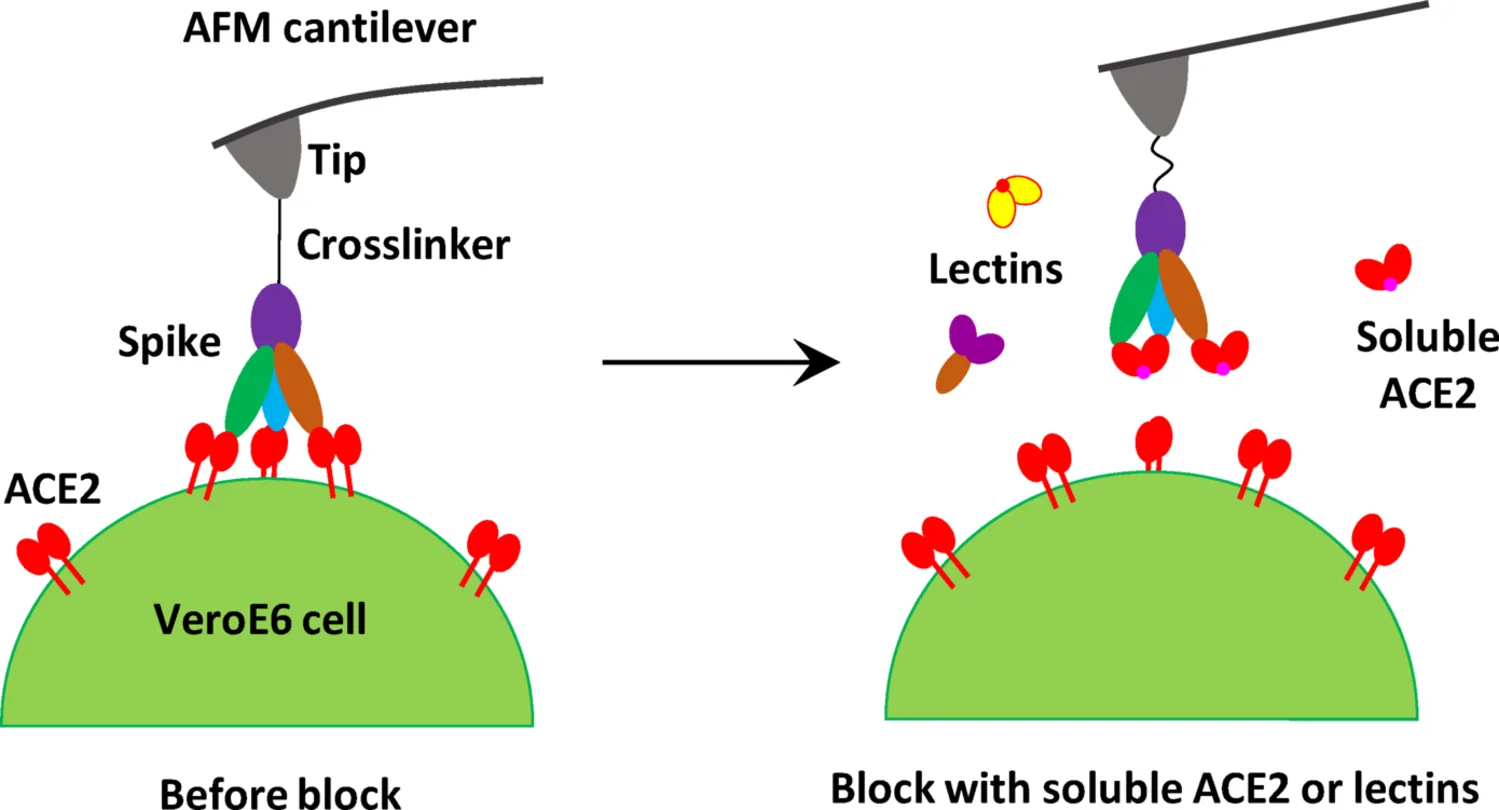
Sketch of the force measurement between the Spike linked with AFM cantilever tip and the ACE2 on the Vero E6 cell (left panel). After adding soluble ACE2 or lectins into the solution (right panel), the binding between the Spike and the ACE2 on the cell surface was inhibited

One of the most important strategies to stop the raging SARS-CoV-2 is to inhibit the initial step of the viral infection, which is the binding between the Spike glycoprotein of the SARS-CoV-2 coronavirus and ACE2 - the receptor expressed on host cells. To elucidate the strength of molecular binding between the Spike and the ACE2 on the single-molecule level, we performed single-molecule force spectroscopy (SMFS) experiments (Fig. 1, left panel).

For these experiments, we functionalized the AFM cantilever tips with SARS-CoV-2 Spike or its mutant. AFM cantilever tips were at first amino-functionalized using the gas phase method for reaction with APTES ((3-Aminopropyl)triethoxysilane). Afterwards, the cantilevers were pegylated with polyethylene glycol (PEG) linker with two different end groups: N-hydroxysuccinimide (NHS) and maleimide. Subsequently, cantilevers were further functionalized with thiol-trisNTA and finally conjugated with His-tagged protein.

Wild type Vero E6 cells endogenously expressing the ACE2 receptor at high level were grown on culture dishes (diameter: 35 mm, height: 10 mm, Greiner Bio-One GmbH, Cat.-No. 627 160) using DMEM containing 10% FBS, 500 units/ml penicillin and 100 µg/ml streptomycin, at 37 °C with 5% CO_2_. For AFM measurements, the cell density was adjusted to about 10-30% confluency. Before the measurements, the growth medium was replaced by physiological HEPES buffer containing 140 mM NaCl, 5 mM KCl, 1 mM MgCl_2_, 1 mM CaCl_2_, and 10 mM HEPES (pH 7.4 with NaOH) supplemented with 50 µM NiCl_2_.

Consecutive force-distance cycle measurements were performed, in which Spike: ACE2 bond formation was allowed upon approaching the Spike-loaded cantilever towards the cells. By subsequently moving the AFM cantilever away from the cellular surface with defined speed, the formed Spike: ACE2 bonds were mechanically pulled with an external force that increased over time until they were broken. At rupture, the dissociation forces were recorded and used to construct the experimental probability density functions of forces. As we reported previously, the Spike trimer can act as a molecular caliper, forming up to three individual bonds with three different ACE2 receptors (Zhu et al. 2022). Upon pulling, they dissociated either sequentially or simultaneously. To decipher the dynamic features of the Spike: ACE2 bonds, we varied the pulling speed in our SMFS experiments. Individual dissociation forces were plotted versus their loading rate (pulling speed times effective spring constant of molecules and AFM cantilever). SMFS data of RBD and the single forces of the Spike protein overlapped and showed similar fitting results using the Bell-Evans model for the kinetic off-rate, i.e. *k*_off_ = 3.2 × 10^− 4±0.5^ s^− 1^ for RBD and *k*_off_ = 5.2 × 10^− 4±0.4^ s^− 1^ for the Spike protein. Equally similar were kinetic on-rates, i.e. *k*_on_ = 1.0 × 10^5±0.08^ M^− 1^s^− 1^ for RBD and *k*_on_ = 0.83 × 10^5±0.04^ M^− 1^s^− 1^ for the Spike trimer. The kinetic on-rates, *k*_on_, were computed from the increase of the binding activity P(t) with the tip-cell surface contact time t_c_, by approximating with pseudo first-order kinetics according to P = A (1 - exp(-(t_c_ - t_0_)/τ ), and calculated using *k*_on_ = 1 / τ C_eff_ (C_eff_, effective concentration of molecules coupled to the AFM tip). Affinities computed from the kinetic rates resulted in a K_D_=3.2 × 10^− 9±0.5^ M for RBD and K_D_ = 6.2 × 10^− 9±0.5^ M for a single RBD of the Spike trimer.

Next, we elucidated differences in the ACE2 binding kinetics of the Delta and the Omicron (B.1.1.529 variant) Spike proteins as compared to the Wuhan isolate. The binding strengths of the Delta Spike trimer were higher and displayed almost 10-fold lower kinetic off-rates (for Delta Spike *k*_off_ = 4.4 × 10^− 5±0.3^ s^− 1^) when compared to that obtained with the Wuhan strain (*k*_off_ = 5.2 × 10^− 4±0.4^ s^− 1^). Thus, the markedly increased binding of the Delta variant might contribute to its increased infectivity. Intriguingly, the kinetic off-rate of the Omicron (B.1.1.529) Spike trimer was about 100-fold lower (*k*_off_ = 4.2 × 10^− 6±0.1^ s^− 1^) than that of the Wuhan strain. In contrast, with both tested Spike variants we did not observe differences in the kinetic on-rates (for Delta Spike 0.89 × 10^5±0.1^ M^− 1^s^− 1^, for Omicron (B.1.1.529) Spike 0.74 × 10^5±0.1^ M^− 1^s^− 1^).

## Inhibition by soluble ACE2

Very recently, great efforts have been put into the development of prophylactic (vaccines) and therapeutics measures, such as drugs (antivirals) or neutralizing antibodies. However, new viral variants with mutations in the Spike protein are constantly arising and bear the risk of higher transmissibility or more severe illness. Recent studies reveal that some of the mutations may escape the neutralization by Spike-specific antibodies, which may potentially reduce the efficacy and reliability of antibody therapies and vaccine development. Therefore, it is essential to explore ligands that may act as efficient antivirals for mutated variants and inhibit their binding to host cells. Finding of effective inhibitors relies on the understanding of the detailed process of the interaction between the Spike mutants and the cell receptors. We explored the inhibitory effect of the soluble ACE2 on the binding between Spike variants of concern and receptors on living cells (Fig. 1, right panel).

For these experiments, we used Vero E6 cells and functionalized AFM cantilever tips with SARS-CoV-2 Spike described in the previous section. All force-distance curves were recorded at room temperature using a PicoPlus 5500 AFM setup and the software PicoScan 5 (Agilent Technologies, Chandler, AZ, USA) with the assistance of a CCD camera for localization of the cantilever tip on selected cells. The sweep range was fixed at 3000 nm, and the sweep rate was set at 1 Hz. For each cell, at least 100 force–distance cycles with 2000 data points per cycle and a typical force limit of about 30 pN were recorded. The unbinding event was identified by local maximum analysis using a signal-to-noise threshold of 2. The binding activity was calculated as the percentage of curves showing unbinding events. For example, if 150 curves from 1000 measured curves show unbinding events, the binding activity amounts to 15%.

**Fig. 2:**
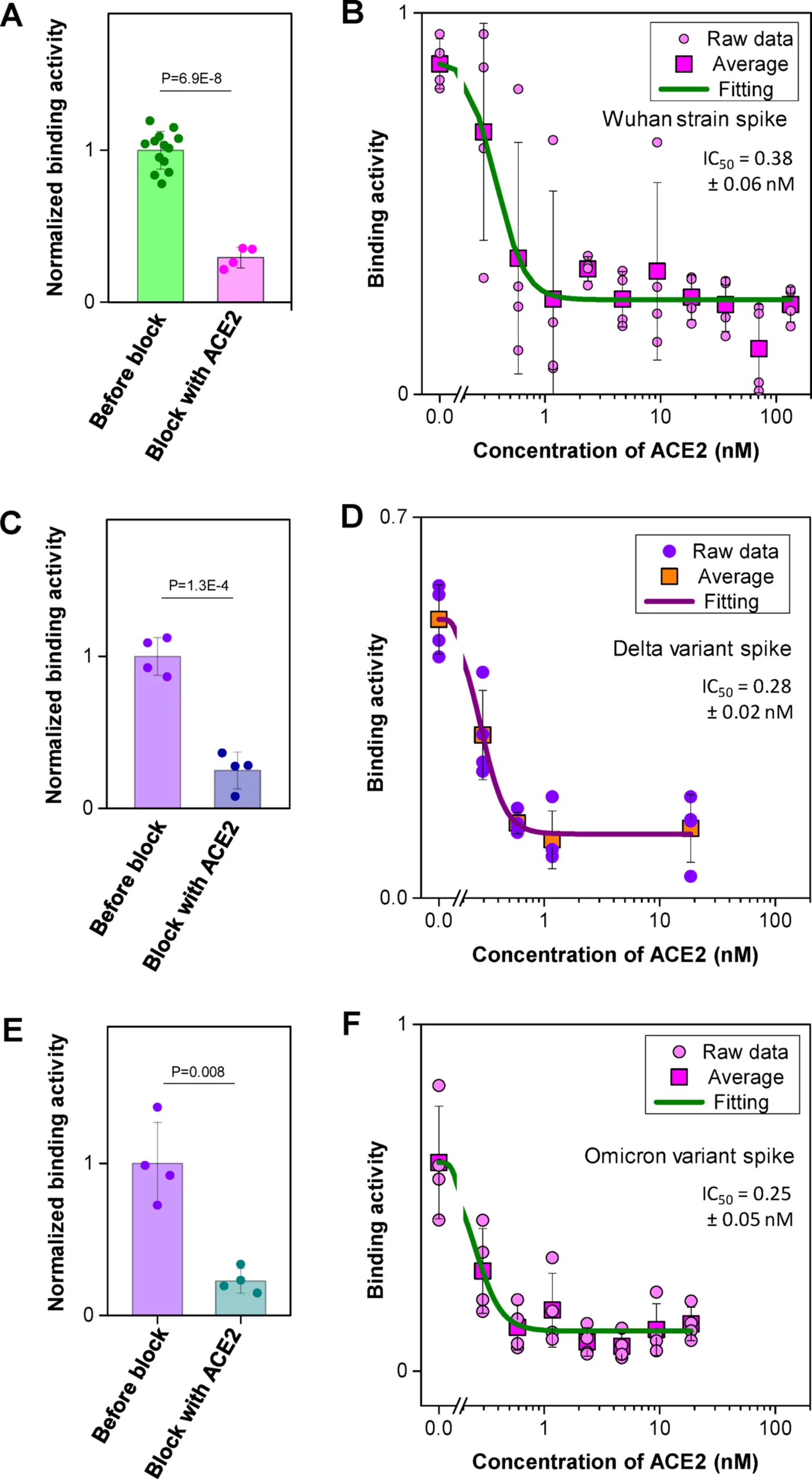
Soluble ACE2 molecules block the binding between Spike trimer on the AFM tip and ACE2 on the surface of Vero E6 cells. (**A**) Blocking experiment for Wuhan strain Spike trimer (*n* = 13 cells before block and 4 cells after block). (**B**) Concentration-dependent blocking of Wuhan strain Spike trimer with soluble ACE2 (*n* = 4 cells). (**C**) Blocking experiment for Delta variant Spike trimer (*n* = 4 cells). (**D**) Concentration-dependent blocking of Delta variant Spike trimer with soluble ACE2 (*n* = 4 cells). (**E**) Blocking experiment for Omicron variant Spike trimer (*n* = 4 cells). (**F**) Concentration-dependent blocking of Omicron variant Spike trimer with soluble ACE2 (*n* = 4 cells). In all panels, the error bars indicate SD and the two-sided Welch’s t-Test is used

Clinical-grade soluble recombinant human ACE2 (amino acids 1-740) for the inhibition experiments was produced by Polymun Scientific (contract manufacturer) from CHO cells according to Good Manufacturing Practice guidelines and formulated as a physiological aqueous solution. Soluble ACE2 was added into the measurement solution at 18.5 nM, which significantly blocked Spike binding to host cell membranes as bi-valent inhibitor (Fig. 2, left panels, two-sided Welch’s t-Test). Moreover, we determined the IC_50_ values by quantifying the inhibition of Spike-ACE2 interactions at the single-molecule level using SMFS. Binding activities were obtained at various inhibitor concentrations, and the fraction of binding events was fitted to a dose-response curve to derive IC_50_ values. The IC_50_ value for the inhibition of Wuhan strain Spike by soluble ACE2 was 0.38 nM (Fig. 2B), which is markedly superior to the dissociation constant derived for single RBD: ACE2 bond of Wuhan strain Spike (6.2 nM). For Delta variant Spike, the IC_50_ value of inhibition by soluble ACE2 was determined as 0.28 nM (Fig. 2D), which is slightly lower than the dissociation constant for single RBD: ACE2 bond of Delta Spike (0.5 nM). The IC_50_ value of inhibition of 0.25 nM (Fig. 2F) found for Omicron variant Spike was significantly higher than the equilibrium dissociation constant for single RBD: ACE2 bonds of Omicron Spike (0.06 nM). Interestingly, previous structural and biophysical studies (Zhu et al. 2022) have reported that the Omicron Spike has a more compact structure and less flexibility in terms of the distance between two RBD arms. This compactness may allow simultaneous binding of two RBDs with a soluble ACE2 dimer to induce molecular strain, thereby reducing the IC_50_. Of note, soluble ACE2 can effectively neutralize SARS-CoV-2 and its variants’ binding to host cells and is currently undergoing clinical tests for the treatment of SARS-CoV-2.

## Inhibition by soluble lectins

Lectins play essential roles in various biological processes, including immune responses and vascular regulation, and also function as endogenous receptors for a wide range of human pathogens. Glycosylation of viral proteins is a key mechanism that facilitates proper protein folding while simultaneously shielding immunogenic epitopes from host immune surveillance. To establish this glycan shield, viruses hijack the host glycosylation machinery, thereby presenting self-like glycan patterns on their surface. Beyond immune evasion, these glycans can engage with lectin receptors, which are commonly expressed on immune and endothelial cells. As pattern recognition receptors, lectins contribute to viral internalization and dissemination. This raises the possibility that targeting lectin–glycan interactions could serve as a viable antiviral strategy, including for coronaviruses.

**Fig. 3:**
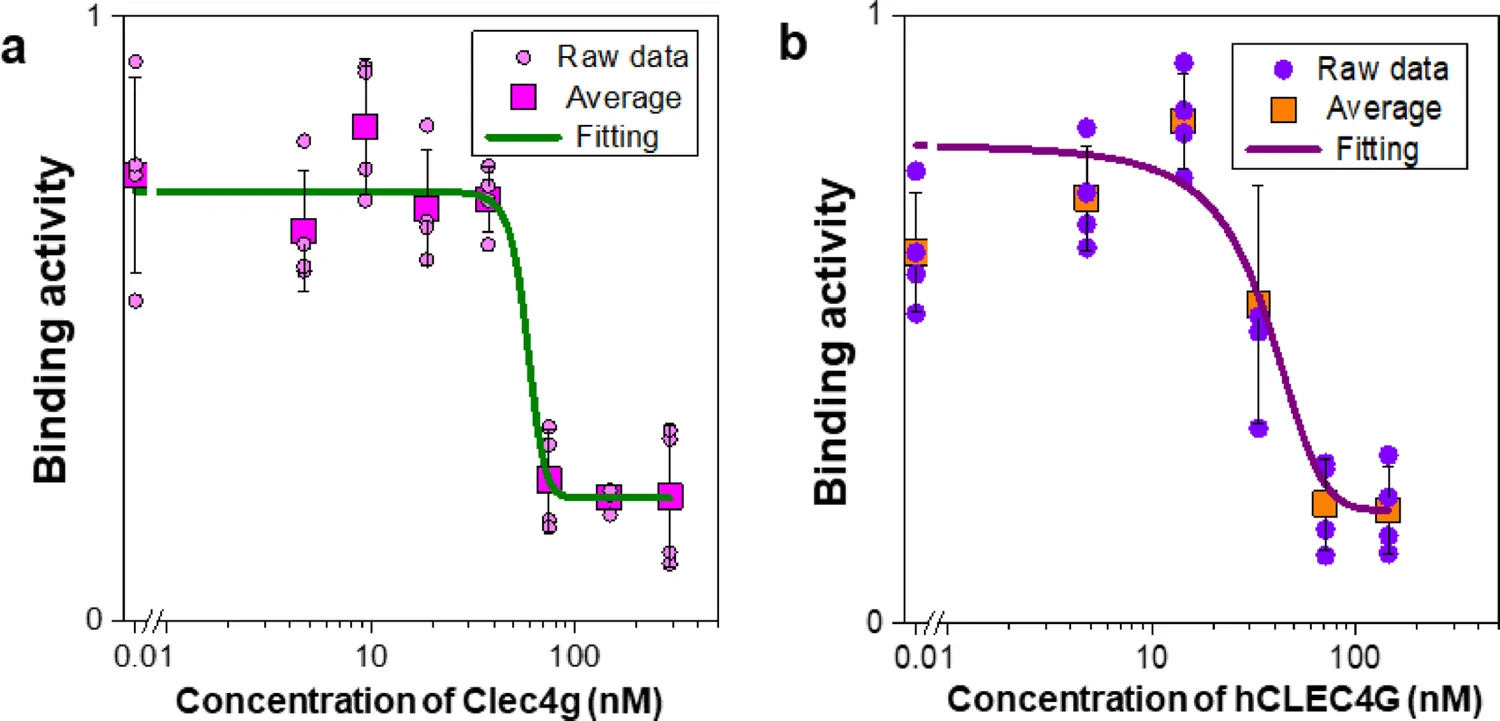
Lectin molecules block the binding between Spike trimer on the AFM tip and ACE2 on the surface of Vero E6 cells. (**A**) Concentration-dependent blocking of Wuhan strain Spike trimer with mouse lectin Clec4g (*n* = 4 cells). (**B**) Concentration-dependent blocking of Delta variant Spike trimer with human lectin hCLEC4G (*n* = 4 cells)

To experimentally evaluate this concept, we investigated whether selected lectins could interfere with the binding of the SARS-CoV-2 Spike trimer to ACE2 receptors on the surface of Vero E6 cells. Using SMFS, we tested several lectins and specifically examined the inhibitory effects of murine Clec4g and human CLEC4G (hCLEC4G). Repeated force measurements on Vero E6 cells revealed binding probabilities in the presence or absence of lectins across various concentrations (Fig. 3). While a control lectin showed no measurable reduction in binding probability over the full concentration range, both Clec4g and hCLEC4G substantially inhibited the Spike–ACE2 interaction (Hoffmann et al. 2021).

Quantitative analysis of the inhibition profiles yielded IC₅₀ values of 58 nM for Clec4g and 36 nM for hCLEC4G, confirming strong inhibitory potency. These results demonstrate that glycan-binding lectins can effectively disrupt Spike-mediated host cell attachment in a dose-dependent manner.

In addition, we have also evaluated the antiviral potential of H84T-Banlec, a rationally engineered banana lectin that selectively binds high-mannose glycans, which are abundant on viral proteins but rare on healthy human cells. H84T-Banlec has been shown to exert potent broad-spectrum activity against viruses with high-mannose N-glycans—including HIV-1/2, influenza A and B, Ebola virus, and hepatitis C virus—by interfering with viral attachment, entry, and subsequent replication steps (Swanson et al. 2015).

**Fig. 4:**
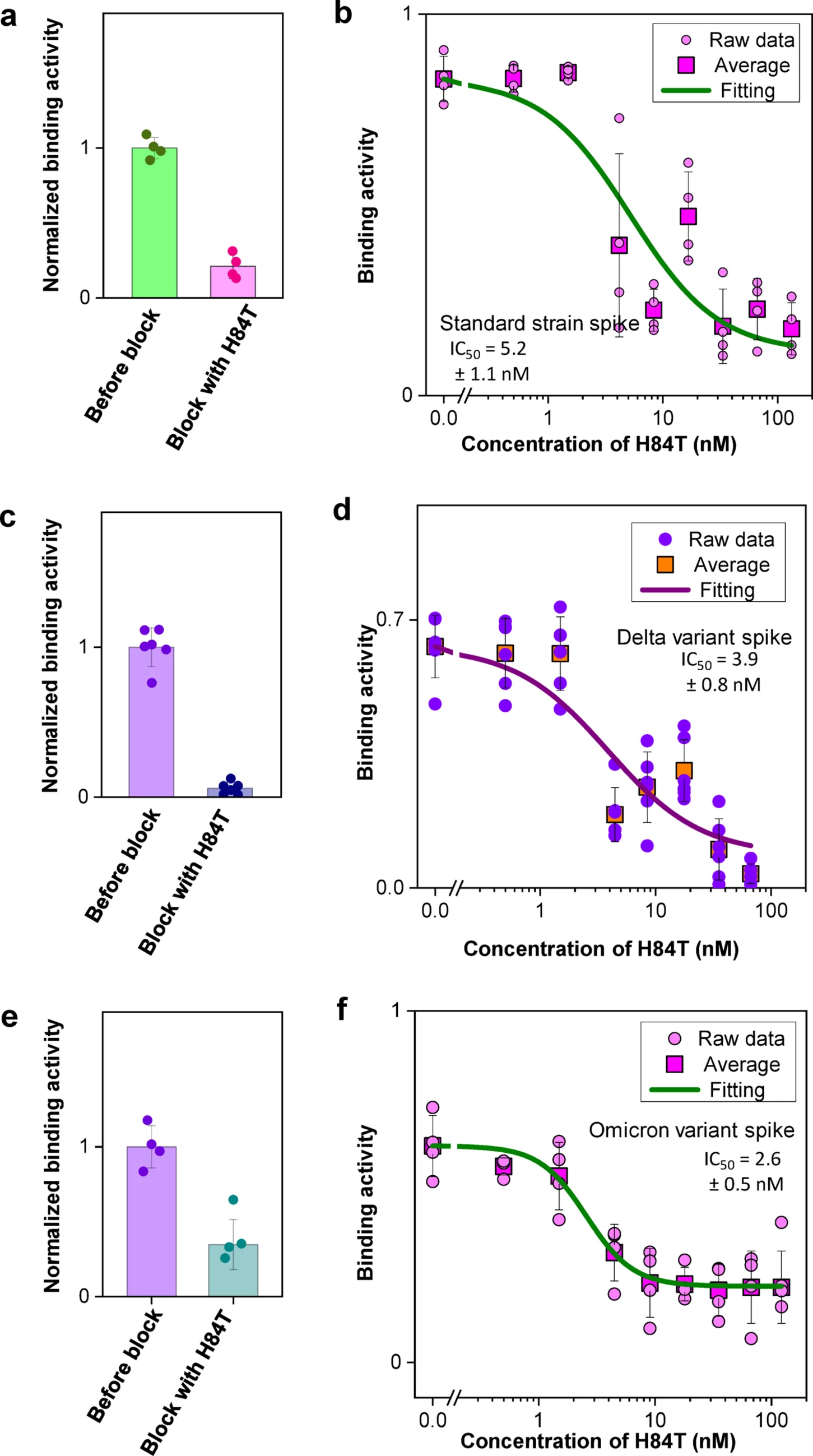
H84T Banlec blocks the binding between Spike trimer on the AFM tip and ACE2 on the surface of Vero E6 cells. (**A**) Blocking experiment for Wuhan strain Spike trimer (*n* = 4 cells before block and 4 cells after block). (**B**) Concentration-dependent blocking of Wuhan strain Spike trimer with Banlec H84T (*n* = 4 cells). (**C**) Blocking experiment for Delta variant Spike trimer (*n* = 6 cells). (**D**) Concentration-dependent blocking of Delta variant Spike trimer with Banlec H84T (*n* = 6 cells). (**E**) Blocking experiment for Omicron variant Spike trimer (*n* = 4 cells). (**F**) Concentration-dependent blocking of Omicron variant Spike trimer with Banlec H84T (*n* = 4 cells)

To assess its inhibitory effect on SARS-CoV-2 Spike binding, we performed concentration-dependent SMFS assays with H84T-Banlec in solution and determined its IC₅₀. Figure 4 illustrates the inhibitory effects of H84T-Banlec across multiple SARS-CoV-2 Spike variants, including the Wuhan strain, Delta, and Omicron. Notably, robust inhibition was observed across all tested variants, supporting the potential of glycan-targeting approaches as a broad-spectrum antiviral strategy (Chan et al. 2022).

H84T-Banlec demonstrated potent, concentration-dependent inhibition of Spike–ACE2 binding across SARS-CoV-2 variants, with IC₅₀ values in the low nanomolar range for Wuhan strain (5.2 ± 1.1 nM), Delta (3.9 ± 0.8 nM), and Omicron (2.6 ± 0.5 nM) Spike proteins. Its bivalent nature likely enhances avidity through multivalent engagement with the trimeric Spike. Notably, these SMFS-derived IC₅₀ values closely aligned with EC₅₀ values obtained from plaque reduction and cytopathic effect assays, further validating the functional relevance of the single-molecule measurements [11]. Here, IC_50_ (half-maximal inhibitory concentration) represents the inhibitor concentration required to suppress 50% of the single-molecule Spike-ACE2 binding events, while EC_50_ (half-maximal effective concentration) refers to the concentration yielding 50% of the maximal antiviral effect in cell-based assay. The strong agreement between IC_50_ and EC_50_ indicates that the single-molecule measurements correspond well with functional antiviral activity at the cellular level, emphasizing the biomedical and physiological relevance of this single-molecule approach.

Consistent with this, cell-based assays confirmed broad antiviral activity against multiple coronaviruses, including SARS-CoV-2 variants, supporting glycan-targeting as a robust, broad-spectrum antiviral strategy.

## Conclusions

Atomic force microscopy-based single-molecule force spectroscopy (AFM-SMFS) provides a powerful approach to quantitatively characterize the interactions between SARS-CoV-2 Spike proteins and the ACE2 receptor at the single-molecule level. By enabling the direct measurement of binding forces and kinetic parameters under physiologically relevant conditions, this methodology affords critical insights into the molecular mechanisms underlying viral attachment and entry. In particular, AFM-SMFS reveals variant-dependent changes in dissociation kinetics, such as slow kinetic off rates, and enables single-molecule quantification of inhibitor potencies (IC_50_) as well as elucidation of multivalent inhibition mechanisms of glycan-binding lectins, thereby complementing and extending information obtained from bulk assays.

In this review, we have illustrated the application of SMFS in assessing molecular inhibitors that disrupt the Spike–ACE2 interface. Soluble ACE2, acting as a decoy receptor, demonstrated highly potent inhibitory activity against multiple SARS-CoV-2 variants, achieving sub-nanomolar IC₅₀ values. Glycan-binding lectins, including murine Clec4g, human CLEC4G, and the engineered H84T-Banlec, similarly displayed substantial inhibition by targeting conserved glycan epitopes on the Spike protein, also yielding IC₅₀ values in the low nanomolar range.

These inhibitors consistently maintained efficacy across several clinically relevant SARS-CoV-2 variants, including the Delta and Omicron strains, reinforcing the therapeutic potential of targeting structurally conserved sites such as receptor-binding and glycan-rich domains. Importantly, the strong correlation between single-molecule IC₅₀ values obtained from SMFS experiments and EC₅₀ values derived from cell-based antiviral assays further supports the biological relevance of this biophysical approach.

Collectively, these findings highlight the utility of AFM-SMFS not only for mechanistic characterization of virus–host interactions but also as an effective biophysical platform for screening and validating potential antiviral inhibitors. Given the continued emergence of viral variants capable of immune evasion, therapeutic strategies focused on conserved structural features of the Spike protein remain particularly promising. Future integration of single-molecule approaches into antiviral discovery efforts could facilitate the identification and optimization of inhibitors capable of broadly counteracting SARS-CoV-2 variants and other viral pathogens.

## Supplementary Information

Below is the link to the electronic supplementary material.

Supplementary Material 1
